# A Versatile Model of Microfluidic Perifusion System for the Evaluation of C-Peptide Secretion Profiles: Comparison Between Human Pancreatic Islets and HLSC-Derived Islet-Like Structures

**DOI:** 10.3390/biomedicines8020026

**Published:** 2020-02-07

**Authors:** Yonathan Gomez, Victor Navarro-Tableros, Ciro Tetta, Giovanni Camussi, Maria Felice Brizzi

**Affiliations:** 1 Unicyte AG, Molecular Biotechnology Center (MBC), University of Turin, Via Nizza, 52, 10126 Turin, Italy; yonathan.gomez@fmc-ag.com; 2 2i3T Società per la Gestione Dell’incubatore di Imprese e per il Trasferimento Tecnologico Scarl, University of Turin, 10126 Turin, Italy; victor.navarro@2i3t.it; 3 Unicyte AG, Unicyte srl, Via Lugaro, 15, 10100 Turin, Italy; ciro.tetta@fmc-ag.com; 4 Department of Medical Sciences, University of Turin, Corso Dogliotti 14, 10126 Turin, Italy; mariafelice.brizzi@unito.it

**Keywords:** microfluidic, perifusion, C-peptide, human pancreatic islets, obese, T2DM, HLSC-ILS

## Abstract

A robust and easy-to-use tool for the ex vivo dynamic evaluation of pancreatic islet (PI) function is essential for further development of novel cell-based therapeutic approaches to treating diabetes. Here, we developed four different glucose perifusion protocols (GPPs) in a microfluidic perifusion system (MPS), based entirely on commercially available components. After validation, the GPPs were used to evaluate C-peptide secretion profiles of PIs derived from different donors (healthy, obese, and type 2 diabetic) and from human liver stem-cell-derived islet-like structures (HLSC-ILS). Using this device, we demonstrated that PIs derived from healthy donors displayed a physiological C-peptide secretion profile as characterized by the response to (a) different glucose concentrations, (b) consecutive pulses of high-glucose concentrations, (c) a glucose threshold ranging from 5–8 mM, and (d) a constant high-glucose perifusion in a biphasic manner. Moreover, we were able to detect a dysregulated secretion profile in PIs derived from both obese and type 2 diabetes mellitus (T2DM) donors. Finally, we also evaluated the kinetic secretion profiles of HLSC-ILS, demonstrating that, nonetheless, with a lower amplitude of secretion compared to PI derived from healthy donors, they were already glucose-responsive on day seven post-differentiation. In conclusion, we have provided evidence that our MPS is a versatile device and may represent a valuable tool to study insulin-producing cells in vitro.

## 1. Introduction

A deep knowledge of insulin-producing β-cell function is critical to identify mechanisms accounting for the lack of secretion in order to allow the discovery of potential targets which could be exploitable in a clinical setting. Unravelling the relationship between glucose stimulus and the production of insulin represented a substantial step towards understanding both normal and impaired functions of pancreatic islets (PI). Independent of the source of insulin-producing cells (either enzymatically isolated islets [1], β-cell lines [2], or stem-cell-derived islet-like structures [3]), two methodologies (based on the format by which the stimuli is provided: static or dynamic) have been used to study insulin secretion in vitro [4]. The static glucose-stimulated insulin secretion (SGSIS) method consists of exposing insulin-producing cells to different glucose concentrations (or other secretagogues) under static conditions during specific time intervals (in general of 1–2 h each), and quantifying the release of insulin/C-peptide. Although it is a fairly simple method and does not require specialized devices, it has two essential limitations: (1) the static batch-type environment substantially differs from the in vivo milieu, and (2) the accumulation of the secreted insulin/C-peptide (or different hormones) may act in an autocrine manner influencing the final result [4]. On the other hand, dynamic glucose-stimulated insulin secretion (DGSIS) protocols were developed to recapitulate the in vivo environment of insulin-producing cells. In addition, dynamic protocols for assessing insulin secretion were present before the static system, just not developed fully due to technical limitations [5,6]. For instance, technical issues related to the use of the whole pancreas (hemolysis and self-digestion of the organ by exocrine enzymes) delayed a broad adoption of the dynamic perifusion methodologies. Later, in the early 1970s, the possibility to enzymatically isolate rat PIs together with the introduction of synthetic materials (plastic chambers and polyethylene tubing) allowed the modernization of the dynamic system which led to milestone discoveries such as the biphasic profile of insulin release [7]. During that period, a major limitation of the improved dynamic system was obtaining flow rates lower than 900 μL/min—as a result the samples were very diluted therefore requiring highly sensitive analytical methods to quantify insulin content. More than two decades ago, the so-called “lab-on-a-chip revolution” was launched in the field of analytical chemistry through the introduction of the microfluidic analysis concept. This allowed the possibility of achieving lesser and more accurate flow rates through smaller devices [8,9] made of materials suitable for cell cultures [10]. On the basis of such technological advances, several groups investigated the functional secretome signature of insulin-producing cells by combining microfluidic systems with electrophoretic immunoassays, which allowed real-time detection of both insulin and calcium oscillations [11,12,13]. More recently, Raman microscopy has been incorporated to visualize molecular structures such as lipids, mitochondria, and nuclei during glucose stimulation [14]. However, since most of these devices are: commercially unavailable, complex, and expensive, their widespread distribution and use are limited. Although efforts have been devoted in developing user-friendly microfluidic devices [15,16], the diabetes research community still needs a flexible, standardized, and less expensive microfluidic perifusion device. Herein, we describe and validate a novel microfluidic perifusion system (MPS) based entirely on commercially available components that can easily be adopted for islet research. By using this MPS we were able to characterize and compare the C-peptide secretion profiles from human PIs derived from different donors (healthy, obese, and type 2 diabetic) and HLSC-ILS (human liver stem-cell-derived islet-like structures) recently described in our lab [3].

## 2. Materials and Methods

### 2.1. Cell Types

HLSC-ILS at day seven post-differentiation were generated as previously described [3]. Human PIs from healthy, obese and diabetic (T2DM) donors were acquired from Prodo Laboratories (Aliso Viejo, CA, USA).

### 2.2. Microfluidic Perifusion System

**Figure 1:**
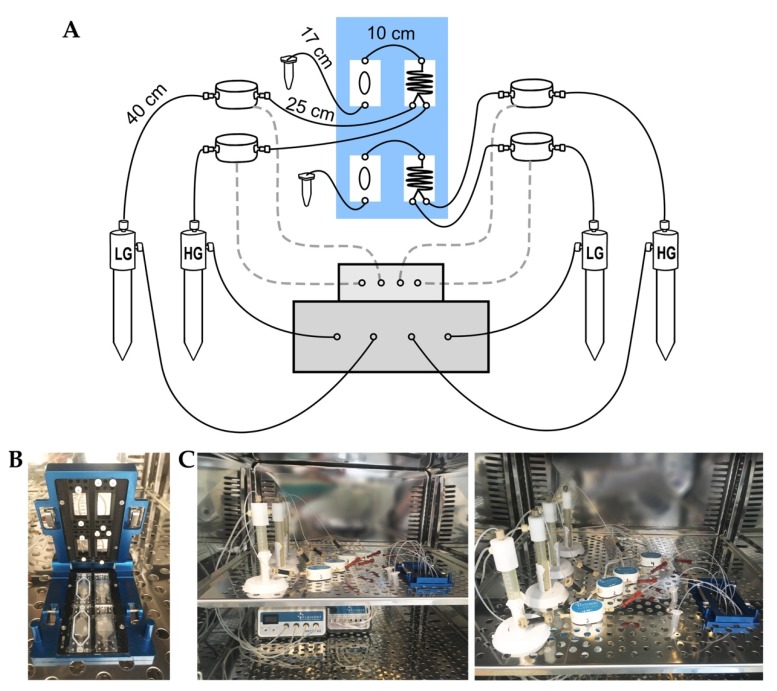
Microfluidic perifusion system (MPS). (**A**) General scheme showing components assembly forming the MPS and the actual length of connection tubing: clamping system (blue box) containing two pairs of mixer and culture chips, flow Board (upper gray box), MFCS-EZ (lower gray box) and four flow rate units and reservoirs connected to mixer chips. (**B**) Picture of Micronit clamping system showing two mixer chips (right) and culture chips (left). (**C**) Picture of the MPS inside the incubator showing the entire system (left) and the reservoirs containing the perifusion solutions connected to the mixer and the culture chips (right).

Our MPS (Figure 1A–C) was designed based on commercially available components. The microfluidic apparatuses were obtained from Fluigent (Okabé, France), while the borosilicate microchips were supplied by Micronit (Overijssel, Nederland). The MPS consists of a vacuum pump (230V-50 Hz, Fluigent, Okabé, France) connected to a pressure-regulator platform (4-channels, 345-mbar, MFCS-EZ, Fluigent, Okabé, France). The pressure-regulator platform was coupled to four independent pressurized reservoirs (Fluiwell-1C, Fluigent, Okabé, France) containing the perifusion solutions and combined with flow-rate sensors (Flow unit M, Fluigent, Okabé, France). Both the pressure-regulator platform and the flow-rate sensors were connected to a flow-rate regulator platform (eight channels, Flowboard; Fluigent; France) which allows real-time monitoring and control of the perifusion solutions using the Maesflo software (version 3.3.4, Fluigent, Okabé, France). The reservoirs containing the perifusion solutions (LG and HG concentration) were connected to a microreactor chip (two inlet and one outlet, 330 mm channel length, 0.3 μL internal volume, Micronit, Overijssel, Nederland) and this was connected to the cell culture chamber made of transparent PET membrane (300 μL internal volume, OCC three-layer layer, Micronit, Overijssel, Nederland). Both the cell culture chambers and microreactor chips were clamped with a four-slot cassette (Fluidic Connect Pro Resealable, Micronit, Overijssel, Netherlands). Liquid connections through the reservoirs to the microchips were made using PTFE tubing (1/16″ OD; 500 μm ID). Compressible perfluoro elastomer ferrules were used to seal PTFE tubing at the inlet and outlet of the chips. All components (except for the vacuum pump and the computer) were kept inside an incubator at 37 °C during the assays.

### 2.3. Glucose Quantification

Output solutions were collected at the end of each stimulatory step and quantified using a commercial glucometer (Accu-Chek Mobile U1 Roche, Mannheim, Germany).

### 2.4. Microfluidic Perifusion of Human PIs and HLSC-ILS

Human PIs were used on the day of arrival from Prodo Laboratories (Aliso Viejo, CA, USA), while HLSC-ILS were evaluated at day seven post-differentiation. Briefly, either 10 IEQ (human PIs) or 400 IEQ (HLSC-ILS) were loaded onto the culture chip in a volume of 100 µL of Krebs buffer (25 mM HEPES, 115 mM NaCl, 24 mM NaHCO_3_, 5 mM KCl, 1 mM MgCl_2_, 2.5 mM CaCl_2_, and 0.1% bovine serum albumin) supplemented with 3 mM of glucose and incubated for 1 h at 37 °C. The MPS was therefore initiated and both the microreactors and empty culture chambers were filled with LG solution (3 mM) at a flow rate of 30 µL/min to confirm the stability of the flow rate and the absence of bubbles or leaks. Afterward, an additional 150 μL of LG solution was added to the middle section of the culture chip and the top layer was carefully placed over it to seal the three-layer chamber (300 μL internal volume, OCC three-layer layer; Micronit, Overijssel, Nederland) avoiding bubble formation. The empty cell culture chambers used for the test were then replaced with the chamber containing human PIs and/or HLSC-ILS, which were placed into the four-slot cassette and closed (Fluidic Connect Pro Resealable, Micronit, Overijssel, Nederland) (Figure 1B). Subsequently, LG was perifused using manual control for 10 min before running the automatic pre-programmed perifusion protocols. Effluents were collected in individual conical tubes and stored at −20 °C to further quantify human C-peptide.

### 2.5. Dynamic Glucose-Stimulated C-Peptide Secretion Assays

Four different GPPs were designed and validated to achieve specific glucose concentrations:

#### 2.5.1. Single Pulse of Glucose and Potassium Protocol (SPGP)

Concentrations of stimulatory solutions were set up at 3 mM for basal glucose (LG), 28 mM for high glucose (HG), and 50 mM for potassium (KCl). The flow rate corresponded to 30 μL/min. The cells were perifused as follows:

1. 20 min at low glucose (LG)_1_;

2. 15 min of transitioning to high glucose solution (LG→HG);

3. 30 min at high glucose solution (HG);

4. 15 min back to low glucose (HG→LG);

5. 10 min at low glucose solution (LG)_2_;

6. 15 min of transitioning to potassium chloride solution (LG→KCl);

7. 15 min of potassium chloride solution (KCl);

8. 20 min back to low glucose (KCl→LG);

9. 10 min at low glucose solution (LG)_3_.

#### 2.5.2. Double Pulse of Glucose Protocol (DPG)

The concentrations of stimulatory solutions were set-up at 3 mM (LG) and 17 mM (HG). Cells were perifused with two consecutive cycles as follows:

First cycle:

1. 20 min at low glucose (LG)_1_;

2. 15 min of transitioning to high glucose solution (LG→HG)_1_;

3. 30 min at high glucose solution (HG)_1_;

4. 15 min back to (HG→LG)_1_;

5. 10 min at low glucose solution (LG)_2_;

Second cycle:

6. 15 min of transitioning to high glucose solution (LG→HG)_2_;

7. 30 min at high glucose solution (HG)_2_;

8. 20 min back to low glucose solution (LG)_3_;

#### 2.5.3. Ascending Ramp of Glucose Protocol (ARG)

The flow-rate ratios of 3 mM (LG) and 28 mM (HG) glucose solutions were adjusted while maintaining the final flow rate constantly at 30 μL/min (Table 1). All steps had a duration of 10 min and were preceded by a transition period of equal duration between different concentrations.

The total duration of the protocol was 140 min. To calculate the flow-rate ratios the following formula was used: (C_1_.V_1_ + C_2_.V_2_)/ (V_1_ + V_2_) = EC where C_1_= 3 mM, C_2_= 28 mM and V_1_ + V_2_ = 300 μL and EC= expected concentration.

#### 2.5.4. Constant High-Glucose Protocol (CHG)

The concentration of stimulatory solutions were set up at 3 mM (LG) and 17 mM (HG). Cells were perifused for 20 min with LG, followed by a 10-min transition period to HG, which was maintained for a further 65 min. Samples were collected every 5 min.

### 2.6. Human C-Peptide Quantification

Human C-peptide was chosen to evaluate and compare the secretion profiles of PIs for the following reasons: (1) it is known to be produced equimolarly to insulin, (2) its secretion provides evidence that stem-cell-derived islet-like structures hold the entire molecular machinery to synthesize insulin.

Human C-peptide concentration was quantified using a ELISA kit (Alpco Diagnosis, Salem, NH, USA) as indicated by the manufacturer’s instruction. The concentrations were calculated using the online data analysis tool “Myassay Ltd”. (Four Parametric Logistic Curves Analysis).

### 2.7. Statistical Analysis

The number of experiments is reported in the Figure legends. All data were analyzed with Prism Software (version 7, GraphPad Software Inc, CA, USA) and presented as mean ± standard deviation (SD). Student’s *t*-test was used for comparison between two groups. When more than two groups were analyzed, ANOVA was used and the Turkey’s multi-comparison tests were applied when appropriate. *p*-values < 0.05 were considered significant. Pearson correlation and the area under the curve (AUC) were calculated based on glucose and C-peptide concentration data respectively.

## 3. Result

### 3.1. Conceptual Design and Assembly of the Microfluidic Perifusion System

The first goal of this study was to design an MPS entirely based on commercially available components (Figure 1A–C). Thus, components from different providers were evaluated in terms of versatility, simplicity, and costs before selecting the French company Fluigent for pressure and flow regulator systems and the Dutch company Micronit Microfluidics for the cell culture chambers, microreactors, and clamping apparatus (see Materials and Methods for technical specifications). Unlike classic peristaltic pumps, the combination of both pressure and flow regulator platforms coupled with flow-rate sensors (Figure 1A,C) allowed the control (either manually or automatically) of flow rates of stimulatory solutions in a highly accurate and stable manner. The perifusion components (pressure and flow regulator, and flow-rate sensors) of our MPS were specifically calibrated to be used with (but not limited to) a Micronit cell culture chamber (Figure 1B) (see Materials and Methods for technical specifications) for the following reasons: (1) its three-layer design allows easy seeding and retrieval of PIs, (2) it has a small internal volume corresponding to 300 μL, and (3) it retains the possibility of using the cell culture membranes (middle layer) up to three times before disposal. However, the standard Micronit cell culture chambers had an important limitation for the purpose of dynamic perifusion of PIs—the lack of a second inlet to allow the influx of two different stimulatory solutions. To overcome this limitation and to avoid the need for culture-chamber customization, we incorporated a microreactor with two inlets and one outlet connected to the standard cell culture chamber (Figure 1A,B).

**Figure 2:**
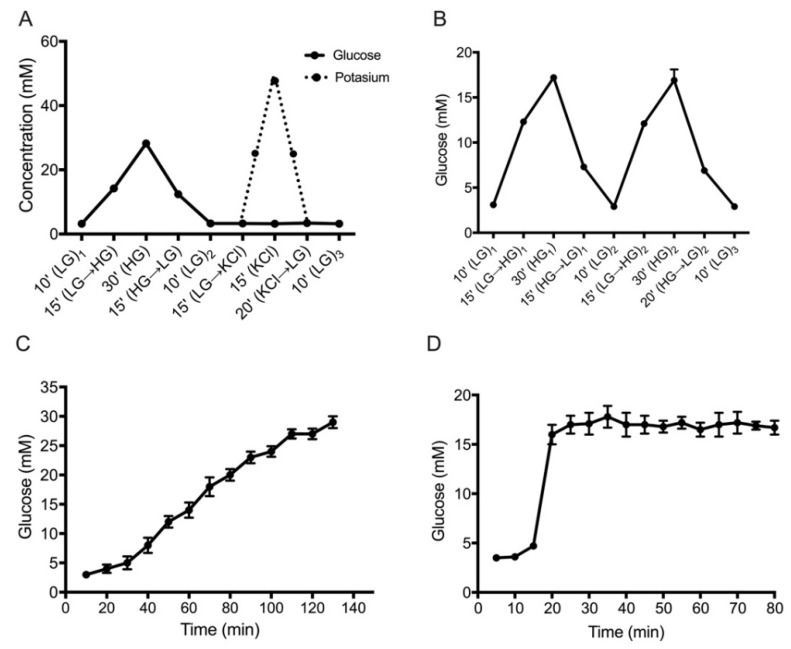
Validation of glucose concentration in four perifusion protocols. (**A**) Single pulse of glucose (filled line; HG = 28 mM) and potassium protocol (dotted line; KCl = 50 mM) SPGP. (**B**) Double pulse of high glucose (HG = 17 mM) protocol (DPG). (**C**) Ascending ramp of glucose concentration from 3–30 mM in 130 min (ARG). (**D**) Constant high-glucose concentration (HG = 17 mM) protocol (CHG). Data are expressed as mean ± SD of *n* = 4 independent experiments. Arrows indicate the direction of transition between stimuli.

By using this approach, we could stimulate PIs not only with two different concentrations of glucose or potassium (Figure 2A–D), but also create an ascending ramp of glucose concentration by fine-tuning the flow ratio of low-glucose (LG) and high-glucose (HG) solutions (Figure 2C). Moreover, to limit shear stress exerted on the PIs and to avoid dilution of the collected samples, we set the flow rate at 30 μL/min, which was the lowest achievable using our system configuration. Additionally, over-dilution in the collected samples was not detected, as demonstrated by the reduced number of human PIs (10 IEQ) required to assess the dynamic glucose-stimulated C-peptide secretion profiles (DGSCS). Furthermore, the flow rate of 30 μL/min was highly stable through the different perifusion protocols due to the flow-control software (FRCM, Fluigent, Okabé, France), which is based on an innovative algorithm that automatically controls the pressures to maintain the flow rate with intra-experimental variations lower than 0.1 μL/min.

### 3.2. Set-Up and Validation of Glucose Perifusion Protocols (GPPs)

Four different GPPs were evaluated to obtain specific glucose concentrations. The time of solution transition (from low to high concentration and vice versa) was determined by collecting output solutions at the end of each stimulatory step and quantifying its glucose concentration with a glucometer (see Materials and Methods). For the single pulse of glucose and potassium protocol (SPGP) (Figure 2A) and for the double pulse of glucose protocol (DPG) (Figure 2B) the transition time was 15 min, while for the ascending ramp of glucose (ARG) (Figure 2C) and the constant high glucose concentration protocol (CHG) (Figure 2D), the time was lowered to 10 min. Also, all protocols provided statistically significant results (*p* < 0.0001) and showed a high rate of reproducibility between independent experiments at the concentration assessed (demonstrated by the Pearson correlation analysis (SPGP: R = 0.9974; DPG: R = 0.9887; ARG: R = 0.9977; CHG R = 0.9761)).

### 3.3. Dynamic Glucose-Stimulated C-Peptide Secretion Profiles of Human PIs and HLSC-ILS

After validation of the perifusion protocols in terms of reproducibility of the glucose concentration determined in each stimulation step, we evaluated whether the MPS could be suitable to assess the C-peptide secretion profiles of both human PI and stem-cell-derived islets (HLSC-ILS). For this purpose, we used human PIs derived from clinically different donors (healthy, obese, and type 2 diabetic) (Table 2), and HLSC-ILS recently described in our lab [3].

**Figure 3:**
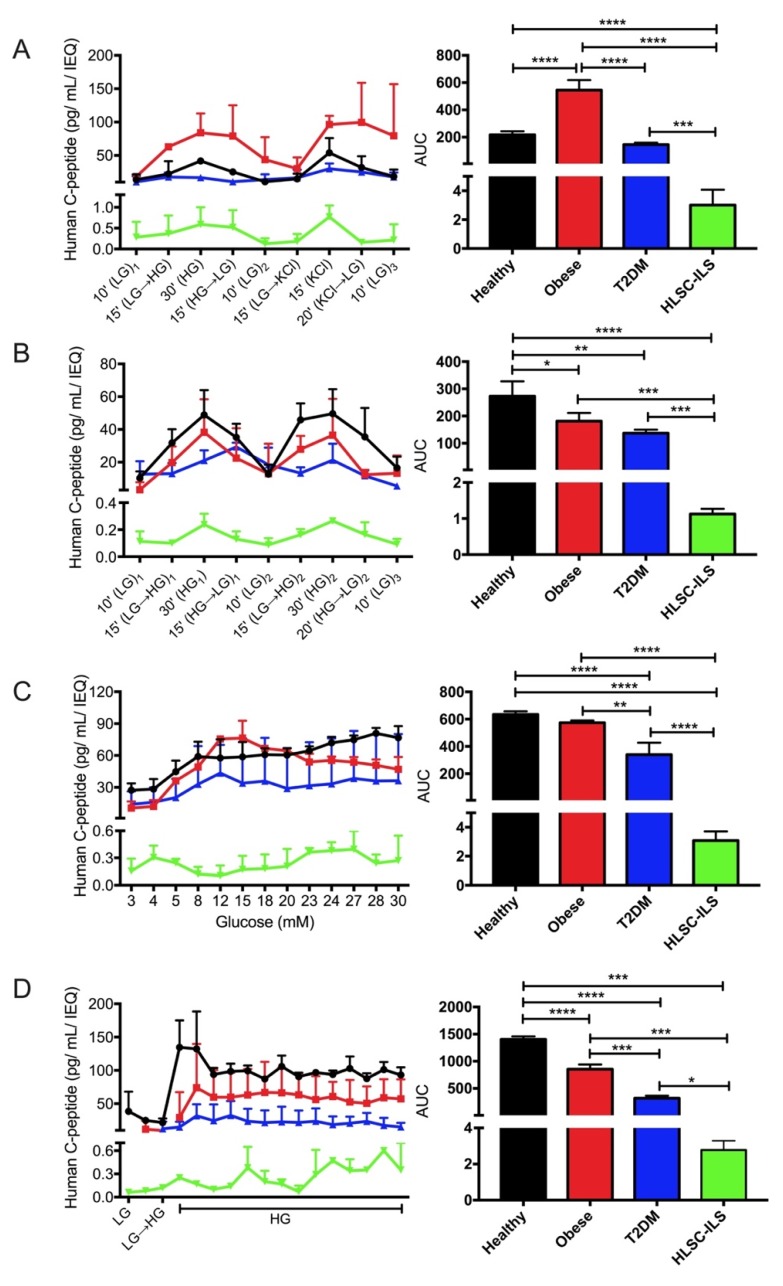
Comparison of dynamic C-peptide secretion profiles (left panels) and areas under the curve (AUCs, right panels) of human PIs derived from different donors and HLSC-ILS. (**A**) Single pulse of glucose (HG; 28 mM) and potassium (KCl; 50 mM) protocol; (**B**) two consecutive pulses of glucose (HG; 17 mM); (**C**) ramp of glucose concentration (3–30 mM); (**D**) constant high-glucose concentration (HG = 17 mM) protocol. Basal concentration of glucose (LG) for all protocols was 3 mM. Results are presented as mean ± SD of PIs from healthy (*n* = 3), obese (*n* = 2), T2DM (*n* = 3) and HLSC-ILS (*n* = 3–5). ANOVA with Turkey’s multi-comparison test was performed; * *p* < 0.05; ** *p* < 0.01; *** *p* < 0.001; **** *p* < 0.0001.

First, we performed the SPGP protocol (Figure 2A) to evaluate C-peptide secretion in response to an HG concentration (28 mM), and the complete degranulation in response to a high-potassium concentration (50 mM). As shown in Figure 3A-left, the healthy and HLSC-ILS secretion profiles were characterized by two main peaks corresponding to the secretion induced by HG (peak 1) and high potassium (peak 2). In addition, when low glucose (LG; 3 mM) was present (LG_1_-LG_2_-LG_3_), C-peptide concentrations returned to the basal state detected during pre-stimulation, therefore indicating that C-peptide secretion was dependent on glucose concentration. Interestingly, the C-peptide secretion profiles of obese and T2DM groups were clearly dysregulated (Figure 3A-left). The amplitude of C-peptide secretion in response to HG stimulus in the obese group was twofold compared to the healthy group and failed to go back to the basal concentration (remained twofold) after KCl stimulation. On the other hand, the T2DM profile displayed a significantly lower (*p* ≤ 0.05) amplitude of response compared to the healthy group, and a lower stimulation index (SI) after HG (2.3 ± 1.1 vs. 3.5 ± 1.3) and KCl (3.4 ± 2.1 vs. 5.1 ± 0.9) stimulation (Figure S1A Complementary Materials).

The DPG protocol (Figure 2B) consists of a narrow low-to-high glucose stimulus (3–17 mM) in order to investigate the possibility that stem -cell-derived islet-like structures might only respond to significant changes in glucose concentrations (3–28 mM). Similar to the previous protocol (SPGP), the kinetic curve of C-peptide secretion of PIs derived from healthy donors and HLSC-ILS (Figure 3B-left) were characterized by two main peaks of secretion during both the HG stimuli (HG_1_ and HG_2_), and by a well-defined basal state during low-glucose concentrations (LG_1_-LG_2_-LG_3_). Nevertheless, the main difference between the two was in the amplitude of the responses (*p* ≤ 0.05) with the healthy PIs being higher. Using this protocol, the dysregulated secretion profiles observed in PIs derived from obese donors did not substantially differ from those of healthy donors. Interestingly, the T2DM profiles showed an overall lower amplitude of responses together with a late response to both HG_1_ and LG_2_ stimuli (Figure 3B-left). In particular, instead of responding to the actual stimuli, they showed a maximum (HG→LG)_1_ and minimum response during the transition states (LG→HG)_2_.

The third protocol applied was the ARG, in which concentrations of glucose ranging from 3–30 mM were perifused and samples were collected every 10 min (Figure 2C). This protocol was performed to investigate glucose sensitivity, i.e., the concentration threshold which triggers the first C-peptide secretion response (defined as SI ≥ 2). As shown in Figure 3C-left, this concentration ranged from 5–8 mM for human PIs independently of its clinical classification (healthy, obese, and T2DM). Interestingly, the kinetic curve (but not the amplitude of response) in both obese and T2DM groups were similar. They reached a peak of secretion between 12–15 mM followed by a sustained decrease until 30 mM of glucose (Figure 3C-left). Conversely, the healthy group showed a transient plateau of C-peptide secretion between 8–20 mM followed by a further increase between 23–28 mM of glucose (Figure 3C-left). In HLSC-ILS the glucose threshold for C-peptide secretion was 4 mM (earlier than in the other groups) and was followed by a reduction in secretion between 5–20 mM of glucose. A trend in restoration of C-peptide secretion was detected from 20–27 mM and a relative reduction at the end of perifusion (28–30 mM).

Finally, the CHG protocol (Figure 2D) was designed to study the well-known biphasic pattern of insulin/C-peptide secretion in insulin-producing cells. It involves perifusing 17 mM of glucose followed by sample collection every 5 min for 65 min. As expected, the secretion profile of healthy PIs was characterized by a rapid increase during the first 10 min of stimulation (first phase), followed by a decreased but sustained secretion of C-peptide (second phase) (Figure 3D-left). Interestingly, the obese group showed a significant (*p* ≤ 0.05) delay in its first phase compared to the healthy group, while in the T2DM group a virtual loss of the first phase was observed. Moreover, similar to our previous protocols, the amplitude of C-peptide secretion was higher in the obese group compared to T2DM. On the other hand, HLSC-ILS displayed a robust first phase of secretion (SI~5) followed by an oscillatory second phase, characterized by peaks of secretion every 15 min (Figure 3D-left).

To better compare the experimental groups (Table 1) and HLSC-ILS, the cumulative area under the curve (AUC) derived from each C-peptide secretion profile was analyzed (Figure 3A–D-right). By using this approach, we noticed that only in the SPGP protocol (Figure 3A-right) (high-glucose concentration: 28 mM), the AUC in the obese group was almost threefold higher compared to the other groups (Figure 3B–D-right). However, with this protocol, no significant differences were observed between the healthy and T2DM groups. Conversely, in the DPG protocol (Figure 2B) the AUC of the C-peptide secretion profile in the healthy group was significantly higher than in the other conditions (obese, T2DM, and HLSC-ILS). However, we did not find differences between obese and T2DM on analyzing this protocol (Figure 3B-right). With the ARG protocol (Figure 2C) we were able to successfully differentiate healthy, T2DM, and HLSC-ILS. In addition, differences between obese and T2DM, but not between healthy and obese groups, were detected (Figure 3C-right). Finally, Figure 3D-right demonstrates that the CHG protocol (Figure 2D) was the most suitable protocol by which to compare different groups. In fact, using this protocol, a statistically significant difference between healthy and other groups (obese, T2DM, and HLSC-ILS) and between obese and T2DM was observed. This suggests that a more frequent sample collection could provide a better resolution of the secretion profiles, and could allow the identification of critical variations in the biphasic response.

## 4. Discussion

The assessment of human PI functionality before transplantation could improve the patient’s clinical outcome. A reliable and highly reproducible perifusion system for the assessment of β-cell function would be highly appreciated by the islet research community. In this study, we reported the design and validation of an MPS built entirely on commercially available components. This MPS allows the automatization of the perifusion protocols, an easy and simple loading and retrieval of PIs due to culture chips, a reproducible and stable flow rate without the need for pre-calibration, and the employment of components that can easily be set-up inside a standard cell incubator. An additional value of the MPS described herein is the possibility to design several highly reproducible GPPs to investigate specific aspects of PI function. As a validation model to evaluate the feasibility of our MPS, C-peptide secretion profiles of PIs derived from different donors (healthy, obese, and T2DM) were analyzed for their ability to respond to (1) glucose in a concentration-dependent manner, (2) consecutive HG pulses, (3) an ascending ramp of glucose concentration, and 4) a constant perifusion of glucose. As expected, the C-peptide secretion profiles of PIs derived from healthy donors were characterized by the ability response to (a) different glucose concentrations, (b) consecutive pulses of high-glucose concentrations, (c) a glucose threshold ranging from 5–8 mM, and (d) a constant high-glucose perifusion in a biphasic manner. Such a profile recapitulates some features that have been previously reported for dynamic GSIS assays [15,16,17,18]. When PIs derived from obese donors were evaluated, we found a dysregulated profile characterized by hyper-responsiveness to 28 mM but not to 17 mM of glucose, the failure to go back to basal concentration after KCl-induced depolarization, the failure to maintain glucose-dependent secretion after increasing glucose concentration, and a reduced amplitude of the first secretion phase. Of note, we detected further functional deteriorations when PIs derived from T2DM donors were exposed to the GPPs. In particular, consistent with β-cell dysfunction they showed a lower amplitude in C-peptide secretion, a delayed glucose-response, and a virtually absent first phase of secretion. Similar results have been reported in models of obesity and T2DM both in vitro and in vivo [19,20,21]. Since HbA1C levels were significantly higher in T2DM compared to obese and healthy donors, it can be postulated that such dysfunctional response may reflect the impaired PI function driven by their long-lasting exposure to HG [22,23]. In summary, despite the limited number of human samples analyzed, our MPS could be considered a useful tool to detect differences in the secretion profiles in clinically different donors. Hence, the results on human PIs validate the feasibility of our MPS to study β-cell function. Moreover, this device would also allow to investigate different endocrine cells, such as α-cells, by simply perfusing stimulatory solutions containing lower glucose concentration (1–3 mM) or secretagogues.

Recently, our lab has described a simple charge-based single-step protocol to differentiate human liver stem cells (HLSC) into islet-like structures [3]. These islet-like structures, not only express several genetic markers of β-cell differentiation, but were also glucose-responsive and, most importantly, were able to restore euglycemia in STZ-induced SCID diabetic mice when implanted at day 14 post-differentiation [3]. By using our MPS we were able to characterize the dynamic secretion profile of HLSC-ILS at day seven post-differentiation. The SPGP protocol indicates that HLSC-ILS were able to secrete C-peptide in a concentration-dependent manner when stimulated with 28 mM of glucose and 50 mM of KCl. With the DPG protocol, we demonstrated that HLSC-ILS were also able to secrete the C-peptide in response to consecutive pulses of 17 mM of glucose and to return to the basal state detected during pre-stimulation in the presence of low glucose. Both the kinetics of secretion and the SIs were comparable to those of PIs derived from healthy donors (being the SIs ≥ 2). For a more detailed characterization, two additional perifusion protocols were carried out. The ARG protocol revealed that the glucose threshold for C-peptide secretion in HLSC-ILS occurred earlier (4 mM) than in PIs from healthy donors (5–8 mM). Additional differences were observed between the secretion profiles of healthy PIs and HLSC-ILS by using the CHG protocol. We found that HLSC-ILS displayed a robust first phase of secretion (SI~5) followed by an oscillatory second phase, characterized by peaks of secretion every 15 min. We hypothesize that such oscillations in the second phase of secretion were likely caused by a lack of enough “reserve pool” of secretory granules [19]. These data confirm the fairly immature secretion profile observed before undergoing further maturation after transplantation in diabetic mice [3]. Future studies will be performed to investigate the possibility to induce in vitro maturation of HLSC-ILS by using different culture media or adding differentiation factors [24]. Furthermore, our data provides evidence that the MPS we propose is a valuable device to assess β-cell function, and a useful tool to detect features of the ß-cell secretion profile, otherwise missing in static conditions.

Finally, the novelty of our MPS could be summarized as follows: (a) it is a more versatile, standardized, and less expensive microfluidic perifusion system, which is entirely based on commercially available components; (b) it allows the evaluation of several dynamic aspects of β-cell functionality; (c) it requires a low number of PIs (10 IEQ); (d) it could generate low flow rates (30 uL/min), which avoids sample dilution and limits the detrimental effects of shear stress on PIs, thanks to the combination of a pressure- and flow-regulator platform.

## Figures and Tables

**Table 1:** Flow-rate ratios (µL/min) of the stimulatory solutions (LG and HG) and expected glucose concentration in the ascending ramp of glucose (ARG) protocol.

Flow Rate (LG)	Flow Rate (HG)	Expected Concentration (mM)
30.0	0	3
27.7	2.3	5
21.8	8.2	10
15.8	14.2	15
9.8	20.2	20
3.8	26.2	25
0	30	28

**Table 2:** Islet donor characteristics and comparison between groups. ANOVA with Turkey’s multi-comparison test was performed. Data are expressed as mean ± SD.

	Healthy (*n* = 3)		Obese (*n* = 2)		T2DM (*n* = 3)
Age (years)	36.7 ± 17.7		51 ± 5.6		61.6 ± 7.5
BMI (kg/m^2^)	24.4 ± 1.5		30.4 ± 3.2		29.9 ± 9.5
HbA1C (%)	5.7 ± 0.2		5.1 ± 0.2		6.8 ± 1.3
Groups comparison		Parameter		*p*-values	
Healthy vs. obese		BMI (kg/m^2^)		*p* < 0.01	
Healthy vs. T2DM				*p* < 0.01	
Obese vs. T2DM				n.s	
Healthy vs. obese		HBA1C (%)		n.s	
Healthy vs. T2DM				*p* < 0.001	
Obese vs. T2DM				*p* < 0.001	
Healthy vs. obese		Age (years)		n.s	
Healthy vs. T2DM				n.s	
Obese vs. T2DM				n.s	

## Supplementary Materials

Supplementary materials can be found at https://www.mdpi.com/2227-9059/8/2/26/s1.

## Abbreviations

ARG	Ascending ramp of glucose
AUC	Area under the curve
CHG	Constant high-glucose concentration protocol
DGSCS	Dynamic glucose-stimulated C-peptide secretion
DGSIS	Dynamic glucose-stimulated insulin secretion
DPG	Double pulse of glucose protocol
GPPs	Glucose perifusion protocols
HG	High glucose
HLSC	Human liver stem-cell
HLSC-ILS	Human liver stem-cell-derived islet-like structures
IEQ	Islet equivalent
KCl	Potassium chloride
LG	Low glucose
MPS	Microfluidic perifusion system
PIs	Pancreatic islets
STZ	Streptozotocin
SCID	Severe combined immunodeficient mice
SGSIS	Static glucose-stimulated insulin secretion
SPGP	Single pulse of glucose and potassium protocol
T2DM	Type 2 diabetes mellitus
ARG	Ascending ramp of glucose
AUC	Area under the curve
CHG	Constant high-glucose concentration protocol
DGSCS	Dynamic glucose-stimulated C-peptide secretion
DGSIS	Dynamic glucose-stimulated insulin secretion
DPG	Double pulse of glucose protocol
GPPs	Glucose perifusion protocols
HG	High glucose
HLSC	Human liver stem-cell
HLSC-ILS	Human liver stem-cell-derived islet-like structures
IEQ	Islet equivalent
KCl	Potassium chloride
LG	Low glucose
MPS	Microfluidic perifusion system
PIs	Pancreatic islets
STZ	Streptozotocin
SCID	Severe combined immunodeficient mice
SGSIS	Static glucose-stimulated insulin secretion
SPGP	Single pulse of glucose and potassium protocol
T2DM	Type 2 diabetes mellitus
