# Supplementary material for: A Versatile Model of Microfluidic Perifusion System for the Evaluation of C-Peptide Secretion Profiles: Comparison Between Human Pancreatic Islets and HLSC-Derived Islet-Like Structures
Source: Biomedicines. 2020 Feb 7;8(2):26. doi: 10.3390/biomedicines8020026 (PMC7168272; doi:10.3390/biomedicines8020026)
Supplement: Supplementary file 1 [file biomedicines-08-00026-s001.zip › Supplementary Material.docx]

**Supplementary Material**


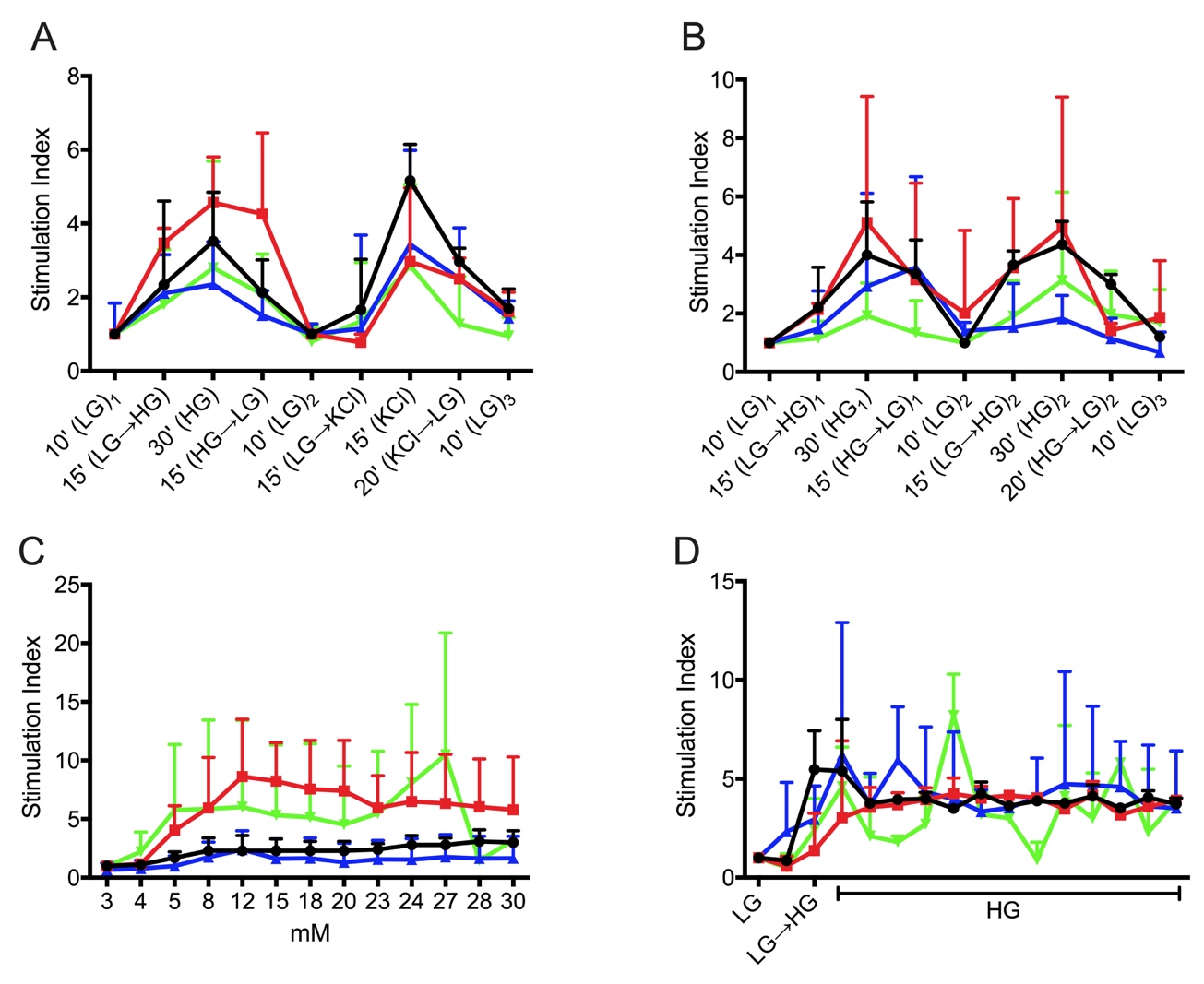


**Figure S1.** Comparison of stimulation indexes (SIs) derived from PIs and HLSC-ILS dynamic C-peptide secretion profiles. SI values for Healthy (black line; n=3), Obese (red line; n=2) and T2DM (blue line; n=3) donors and HLSC-ILS (green line; n=3–5) exposed to (A) Single pulse of glucose (HG;28mM) and potassium (KCl;50mM) protocol; (B) Two consecutive pulses of glucose (HG;17 mM); (C) Ramp of glucose concentrations (3–30 mM); (D) Constant high glucose concentration (HG=17mM) protocol. SIs were calculated by dividing C-peptide levels by the basal release at 3 mM of glucose (LG).
